# Population density, call-response interval, and survival of out-of-hospital cardiac arrest

**DOI:** 10.1186/1476-072X-10-26

**Published:** 2011-04-14

**Authors:** Hideo Yasunaga, Hiroaki Miyata, Hiromasa Horiguchi, Seizan Tanabe, Manabu Akahane, Toshio Ogawa, Soichi Koike, Tomoaki Imamura

**Affiliations:** 1 Department of Health Management and Policy, Graduate School of Medicine, The University of Tokyo, Tokyo, Japan; 2 Department of Health Quality Assessment, Graduate School of Medicine, The University of Tokyo, Tokyo, Japan; 3 Foundation for Ambulance Service Development, Emergency Life-Saving Technique Academy of Tokyo, Tokyo, Japan; 4 Department of Public Health, Health Management and Policy, Nara Medical University School of Medicine, Nara, Japan; 5 Department of Planning, Information and Management, The University of Tokyo Hospital, Tokyo, Japan

## Abstract

**Background:**

Little is known about the effects of geographic variation on outcomes of out-of-hospital cardiac arrest (OHCA). The present study investigated the relationship between population density, time between emergency call and ambulance arrival, and survival of OHCA, using the All-Japan Utstein-style registry database, coupled with geographic information system (GIS) data.

**Methods:**

We examined data from 101,287 bystander-witnessed OHCA patients who received emergency medical services (EMS) through 4,729 ambulatory centers in Japan between 2005 and 2007. Latitudes and longitudes of each center were determined with address-match geocoding, and linked with the Population Census data using GIS. The endpoints were 1-month survival and neurologically favorable 1-month survival defined as Glasgow-Pittsburgh cerebral performance categories 1 or 2.

**Results:**

Overall 1-month survival was 7.8%. Neurologically favorable 1-month survival was 3.6%. In very low-density (<250/km^2^) and very high-density (≥10,000/km^2^) areas, the mean call-response intervals were 9.3 and 6.2 minutes, 1-month survival rates were 5.4% and 9.1%, and neurologically favorable 1-month survival rates were 2.7% and 4.3%, respectively. After adjustment for age, sex, cause of arrest, first aid by bystander and the proportion of neighborhood elderly people ≥65 yrs, patients in very high-density areas had a significantly higher survival rate (odds ratio (OR), 1.64; 95% confidence interval (CI), 1.44 - 1.87; p < 0.001) and neurologically favorable 1-month survival rate (OR, 1.47; 95%CI, 1.22 - 1.77; p < 0.001) compared with those in very low-density areas.

**Conclusion:**

Living in a low-density area was associated with an independent risk of delay in ambulance response, and a low survival rate in cases of OHCA. Distribution of EMS centers according to population size may lead to inequality in health outcomes between urban and rural areas.

## Introduction

Numerous studies have indicated that early initial cardiopulmonary resuscitation (CPR) by laypersons [1,2] and rapid prehospital care by emergency medical service (EMS) providers [3,4] can both substantially increase the survival of out-of-hospital cardiac arrest (OHCA) patients. Over the past several decades, the deployment of trained personnel functioning in an organized EMS system [5] and community-based strategies focusing on early defibrillation with automated external defibrillators (AED) have enhanced the likelihood of successful rescue of OHCA patients [1].

The likelihood of survival of OHCA patients may depend on sociodemographic factors as well as biological and clinical characteristics. One possible social factor that may impede a rapid delivery of EMS is living in a rural area. Geographical barriers in a rural setting, including distance and transportation time, could be crucial factors in the availability of EMS.

With regard to trauma care, increased EMS response time in rural areas has been shown to be a contributing factor to higher mortality rates of critically injured patients from motor vehicle crashes [6]. Several previous studies have suggested a relationship between low population density and low survival rates in cases of OHCA [7-11]. However, these studies were conducted in limited geographic areas or situations.

To make rational decisions about improving the allocation of EMS resources in a whole nation, health policy makers should ideally take into consideration how population density might influence the overall benefit of such implementations. That is, advanced knowledge of the effects of geographic variation on outcomes of OHCA could guide better identification of effective interventions, including equitable access to EMS, and initiatives in the performance of CPR by members of the public. However, such data are not fully available at present.

The aim of the present study was to investigate the relationship between population density, time between emergency call and ambulance arrival, and the survival of OHCA patients in a nationwide setting, using the All-Japan Utstein-style registry database coupled with geographical information system (GIS) data.

## Methods

### Japanese EMS system

The Fire and Disaster Management Agency (FDMA) of Japan supervises the EMS system nationwide. The designated universal emergency call number is 119. This number is directly connected to the neighboring dispatch center with a computerized dispatch system. On receipt of an emergency call, the nearest available ambulance is sent to the incident location. All expenses for EMS are covered by taxes, and patients are not charged.

Generally, an ambulance crew includes three EMS staff members, including at least one emergency life-saving technician, who has undergone extensive training to provide prehospital EMS [1,2,12]. All EMS providers perform CPR in accord with the Japanese CPR guidelines, which are based on the American Heart Association and the International Liaison Committee on Resuscitation guidelines [13,14]. Emergency life-saving technicians are allowed to use several methods, including semiautomated external defibrillators, the insertion of an adjunct airway (esophageal obturator airway or laryngeal mask airway), the insertion of a peripheral intravenous line, and the administration of lactate Ringer solution and epinephrine. Only specially trained emergency life-saving technicians are permitted to insert tracheal tubes [1,2,12].

EMS personnel in Japan are legally prohibited from withholding or terminating resuscitation out of hospital, similar to the case in many countries. Most OHCA patients undergo CPR by EMS providers and are transported to hospitals, except in cases where fatality is clear (e.g. rigor mortis, incineration or decomposition) [1].

### Data source

In January 2005, the FDMA launched a prospective, nationwide, population-based, observational study involving all OHCA patients who received EMS in Japan [1]. EMS personnel in each center recorded the data of OHCA patients with an Utstein-style form [15,16] in cooperation with the physicians in charge of the patients. These anonymous data were electronically sent to the FDMA database server.

The database included the following data: address of the responding EMS center, patients' sex, age, causes of arrest (cardiac or non-cardiac origin), bystander witness status, presence of bystander CPR with or without AED use, the times of the receipt of an emergency call and vehicle arrival at the scene, 1-month survival and neurological outcome 1 month after cardiac arrest defined in terms of the Glasgow-Pittsburgh cerebral performance categories (CPC: good performance, CPC1; moderate disability, CPC2; severe cerebral disability, CPC3; vegetative state, CPC4; or brain death, CPC5) [15,16]. In Utstein-style format, a de facto "brain death" case is considered to be still "alive" if the patient has not been diagnosed with the standard diagnostic criteria for brain death, but is coded as CPC5. The physicians in charge made a diagnosis of cause in collaboration with EMS staff. We defined 'call-response interval' as the interval between emergency call to vehicle arrival at the scene.

The FDMA provided all the anonymous data to our research group. This study was approved by the Institutional Review Board of the Nara Medical University.

### Population and distribution of EMS centers

As of 2005, the population of Japan was approximately 127 million, with several densely populated areas including the Tokyo metropolitan area and the cities of Osaka, Nagoya, Yokohama, Sendai, Fukuoka and Sapporo [17]. The area of the Japanese archipelago is approximately 378,000 km^2^, about two-thirds (257,000 km^2^) of which is uninhabited mountainous terrain.

To assess the relationship between population and distribution of EMS centers, the area of Japan was divided into 359 medical jurisdictions determined according to the Medical Service Law in Japan. The population (variable *X*) and the number of EMS centers (variable *Y*) in each jurisdiction were identified, and the values were plotted on an *X*-*Y *plane. The Pearson's correlation coefficient between populations and the numbers of EMS centers in the 359 jurisdictions was calculated.

### Geographical information

Geographical information system (GIS) is a computer-based approach to the integration and analysis of geographical data [18-21]. Address-match geocoding, one GIS method, is a process that converts full address information to digital spatial data [19].

In the present study, a text-based address of each EMS center was converted to latitude and longitude coordinates. For this procedure, we utilized a website of Japanese address match-geocoding (http://www.geocoding.jp/) established with Google Maps Application Program Interface, powered by Google (Mountain View, CA, US). An address (number and street name) and zone (a town name or zip code) of each responding EMS center derived from the OHCA registry database was compared against the full array of addresses in the foundation database of Google Maps, and a 'match' occurred when the two agreed.

The spatial data were linked with the Population Census data [17], using ArcGIS version 9.3.1. (Environmental Systems Research Institute Inc., Redlands, CA, US). In assessing neighborhood sociodemographic characteristics through the use of ArcGIS, the area of Japan was divided into about 378 thousand squares, each square being 1 km^2^. The population census data were identified for each 1-km^2 ^square area where each EMS center was located, including population density (/km^2^) and the proportion of elderly people aged ≥ 65 years.

### Data Analyses

In the present study, we included OHCA patients whose cardiac arrests were witnessed by bystanders and who received prehospital EMS between Jan 1, 2005 and Dec 31, 2007. Outcome data included overall 1-month survival and neurologically favorable 1-month survival as defined by the Glasgow-Pittsburgh CPC1 or 2. This categorization was determined by a follow-up interview of the physicians in charge by the EMS providers.

Two categories of variables were utilized in the analyses: (i) variables relating to individual patient characteristics and (ii) variables associated with the patients' neighborhood environment. Individual-level patient characteristics included age, sex, causes of arrest (cardiac or non-cardiac origin), presence of bystander CPR with or without AED use, and call-response interval. We also identified the dates of OHCA incidence and divided them into four seasons including spring (Mar-May), summer (Jun-Aug), autumn (Sep-Nov) and winter (Dec-Feb). Variables associated with patients' neighborhood environment were population density, and the proportion of elderly people (aged ≥ 65 years) in each area. Patients were stratified into six population-density groups: very low (<250/km^2^), low (250-999/km^2^), middle-low (1,000-2,999/km^2^), middle-high (3,000-5,999/km^2^), high (6,000-9,999/km^2^), and very high (≥10,000/km^2^) density groups.

Patient characteristics and the neighborhood proportion of elderly people ≥ 65 years were compared between the six population-density groups. We calculated the survival outcomes in each subcategory divided by patient characteristics and patient's neighborhood environment. We performed univariate comparisons of variables using a chi-square test or an analysis of variance (ANOVA) as appropriate. Logistic regression analyses were performed to model the concurrent effects of multiple variables on the outcomes. We first used fixed effect models for logistic regression analyses. Then, we tried to develop hierarchical logistic regression models including site effect. These models included random intercepts for EMS centers in addition to the above-mentioned variables. The threshold for significance was a p-value < 0.05. All statistical analyses were conducted using PASW Statistics version 18.0 (SPSS Inc., Chicago, IL, US).

## Results

We enrolled 101,287 consecutive OHCA patients who had been witnessed by bystanders and who received EMS from 4,729 dispatch centers in Japan between 2005 and 2007. All the addresses of 4,729 centers were successfully geocoded.

**Figure 1:**
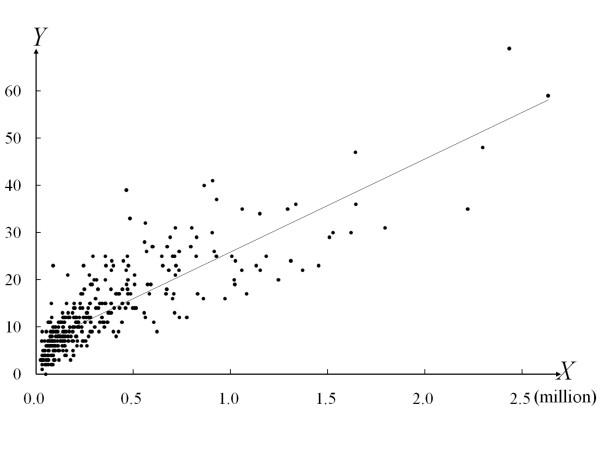
**The correlation between size of population and the number of emergency medical service centers in 359 jurisdictions**. Variable *X *denotes population (million persons), while variable *Y *denotes the number of emergency medical service centers in 359 jurisdictions. *Y *= 19.71*X*+6.16, *R*^*2 *^= 0.708

Figure 1 shows the relationship between population and the number of EMS dispatch centers in 359 jurisdictions. The Pearson's correlation coefficient was 0.841 (p < 0.001), indicating that the distribution of EMS centers was almost in proportion to the size of the population.

**Figure 2:**
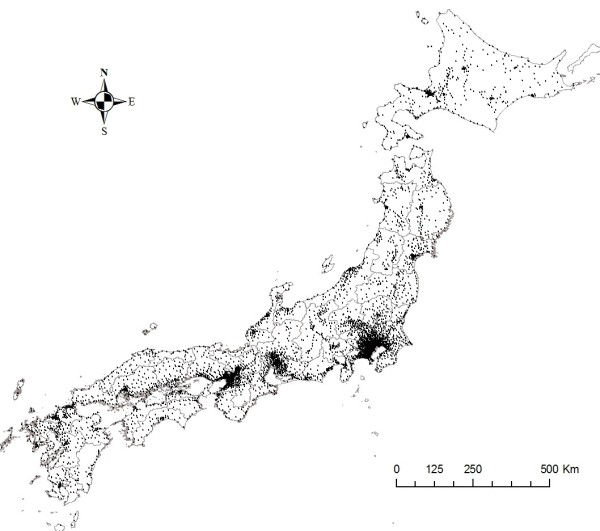
**A map of emergency medical service centers in Japan (47 prefectures)**. Dots indicate locations of emergency medical service centers.

**Figure 3:**
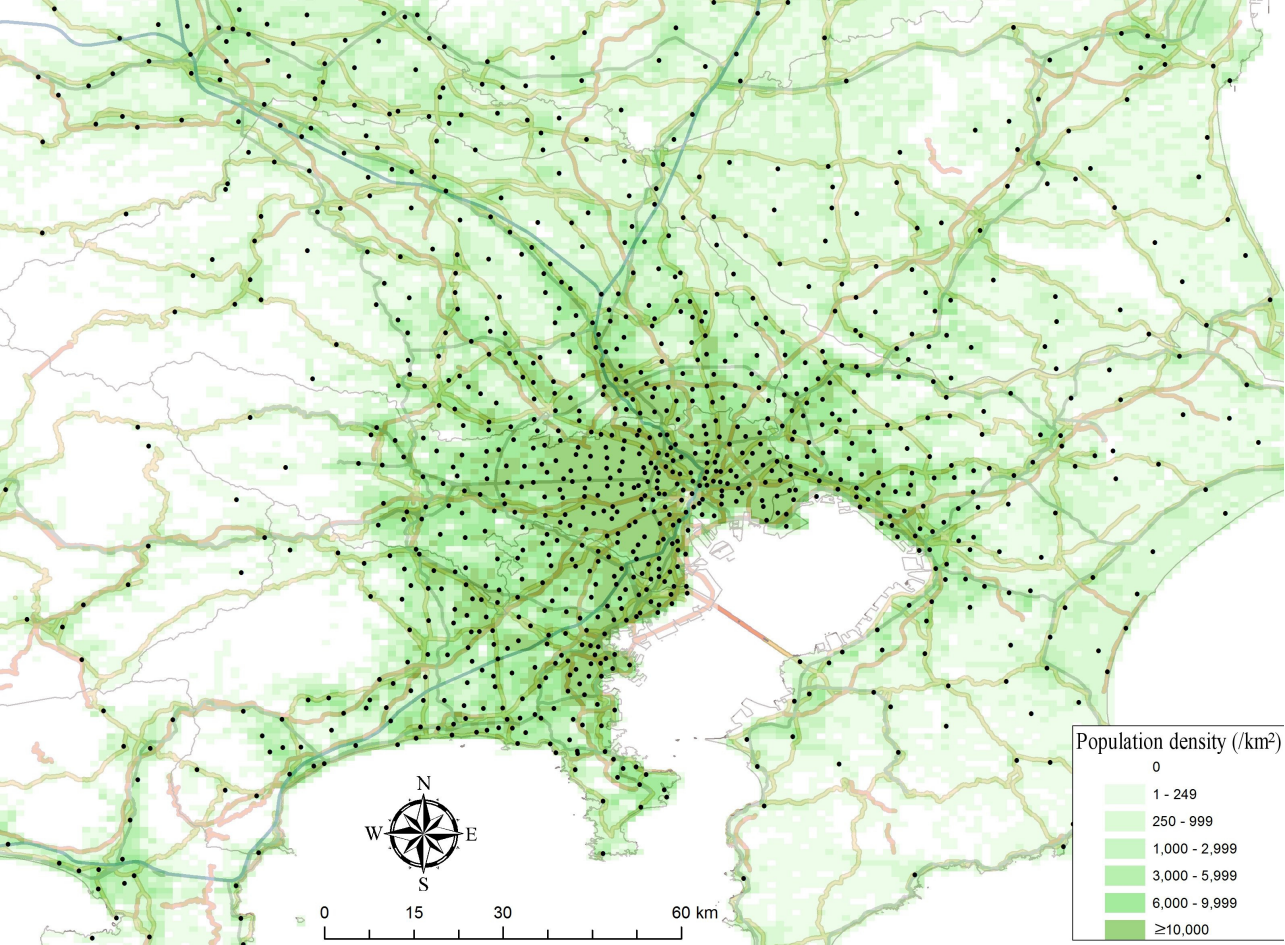
**A map of emergency medical service centers in the Tokyo metropolitan area and surrounding areas coupled with population information**. Dots indicate locations of emergency medical service centers. Green shading indicates population density. White areas are uninhabited. Lines are highways and national roads. Drawn with ArcGIS.

Figure 2 shows a map of EMS centers in Japan, and Figure 3 focuses on EMS centers in the Tokyo metropolitan area and surrounding areas coupled with population information.

Table 1 shows the patient background and neighborhood environment. An ANOVA showed that the average age of patients in lower-density areas was significantly higher than those in higher-density areas (p < 0.001). A chi-square test showed a significant difference in the proportion of elderly people (aged ≥65 years) between the groups; 25.6% in very low-density areas against 18.1% in very high-density areas (p < 0.001).

**Table 1:** Patient characteristics and neighborhood environment in each population-density area

Population density (/km2)	Very low (<250)	Low (250-999)	Middle-low (1,000-2,999)	Middle-high (3,000-5,999)	High (6,000-9,999)	Very high (≥10,000)	p
No. of patients	5,605	14,870	23,057	21,702	16,193	19,860	
Sex (males)	62.6%	61.9%	61.6%	61.7%	61.6%	61.3%	0.587*
Age (Average ± SD, yrs)	73.2 ± 17.0	72.9 ± 17.6	72.2 ± 17.7	71.6 ± 17.8	70.8 ± 18.1	70.5 ± 18.1	<0.001**
Cardiac origin (%)	53.6%	53.6%	54.0%	55.2%	57.2%	58.0%	<0.001*
Occurrence of bystander-initiated CPR	46.4%	45.3%	44.2%	42.8%	41.4%	36.3%	<0.001*
Occurrence of AED use	0.55%	0.35%	0.52%	0.48%	0.59%	0.85%	<0.001*
The proportion of neighborhood elderly people aged ≥65 yrs	25.6%	24.6%	21.9%	20.5%	18.6%	18.1%	<0.001*

CPR: cardiopulmonary resuscitation, AED: automated external defibrillators, SD: standard deviation. *Chi-square tests **Analysis of variance

Table 2 shows the survival outcomes in each subcategory. Seasonal data for 8 patients were missing. Overall, 1-month survival and neurologically favorable 1-month survival occurred in 7,915 (7.8%) and 3,639 (3.6%) cases, respectively. Fewer cases of survival were observed in females (p < 0.001) and higher age groups (p < 0.001). Bystander CPR coupled with AED use showed greater improvements of survival likelihood compared with bystander CPR alone (p < 0.001), but AED was used in only 572 (0.56%) cases. Cases of arrest with cardiac origin showed higher rates of survival than those with non-cardiac origin (p < 0.001). With regard to call-response intervals, 1-month survival rates were 13.2% and 3.6% in the subgroups of ≤ 2 min and ≥11 min, respectively (p < 0.001). Neurologically favorable 1-month survival rates were 8.4% and 1.6% the subgroups of ≤ 2 min and ≥11 min, respectively (p < 0.001). Neighborhoods with a higher proportion of elderly people (≥65 years) were significantly associated with lower 1-month survival (p < 0.001) and neurologically favorable 1-month survival (p < 0.001).

**Table 2:** 1-month survival rates and neurologically favorable 1-month survival rates in each subgroup

	N	1-month survival		Neurologically favorable 1-month survival	
Total	101,287	7,915	(7.8%)	3,639	(3.6%)
Sex					
Male	62,445	5,297	(8.5%)	2,611	(4.2%)
Female	38,842	2,618	(6.7%)	1,028	(2.6%)
Age (yrs)					
≤ 59	20,457	2,473	(12.1%)	1,493	(7.3%)
60 - 69	15,530	1,612	(10.4%)	808	(5.2%)
70 - 79	26,010	1,888	(7.3%)	733	(2.8%)
80 - 89	27,497	1,521	(5.5%)	476	(1.7%)
≥ 90	11,775	419	(3.6%)	128	(1.1%)
First aid by bystander					
None	58,525	3,868	(6.6%)	1,498	(2.6%)
CPR without AED	42,190	3,851	(9.1%)	1,976	(4.7%)
CPR with AED	572	196	(34.3%)	165	(28.8%)
Cause of arrest					
Cardiac	56,188	4,829	(8.6%)	2,640	(4.7%)
Non-cardiac	45,099	3,086	(6.8%)	999	(2.2%)
Seasons					
Summer (June-August)	20,899	1,856	(8.9%)	895	(4.3%)
Autumn (September-November)	23,811	1,966	(8.3%)	929	(3.9%)
Winter (December-February)	30,900	2,188	(7.1%)	973	(3.1%)
Spring (March-May)	25,669	1,904	(7.4%)	842	(3.3%)
Call-response interval (min)					
≤ 2	2,736	361	(13.2%)	229	(8.4%)
3 - 4	17,074	1,899	(11.1%)	931	(5.5%)
5 - 6	30,515	2,770	(9.1%)	1,254	(4.1%)
7 - 8	23,683	1,656	(7.0%)	694	(2.9%)
9 - 10	13,004	707	(5.4%)	301	(2.3%)
≥ 11	14,234	516	(3.6%)	228	(1.6%)
The proportion of neighborhood elderly people ≥65 yrs (%)					
< 15	17,912	1,571	(8.8%)	718	(4.0%)
15 - 25	59,422	4,792	(8.1%)	2,221	(3.7%)
> 25	23,953	1,552	(6.5%)	700	(2.9%)

CPR: cardiopulmonary resuscitation, AED: automated external defibrillators

Table 3 shows the average and 95% confidence intervals (CIs) of call-response interval and outcomes in each population-density group. Comparing between very low- and very high-density areas, call-response intervals were 9.3 [9.1-9.4] min vs. 6.2 [6.1-6.3] min (p < 0.001), 1-month survival rates were 5.4% [4.8-6.0%] vs. 9.1% [8.7-9.5%] (p < 0.001), and neurologically favorable 1-month survival rates were 2.7% [2.3-3.1%] vs. 4.3% [4.0-4.6%] (p < 0.001), respectively.

**Table 3:** Population density, call-response interval and survival of out-of-hospital cardiac arrest

Population density (/km2)	Call-response interval, average [95% CI] (min)		1-month survival, average [95% CI] (%)		Neurologically favorable 1-month survival, average [95% CI] (%)	
Very low (<250)	9.3	[9.1-9.4]	5.4	[4.8-6.0]	2.7	[2.3-3.1]
Low (250-999)	8.4	[8.3-8.5]	6.0	[5.6-6.4]	2.7	[2.4-3.0]
Middle-low (1,000-2,999)	7.7	[7.7-7.8]	7.2	[6.9-7.6]	3.5	[3.3-3.7]
Middle-high (3,000-5,999)	7.2	[7.2-7.3]	8.3	[7.9-8.7]	3.6	[3.3-3.8]
High (6,000-9,999)	6.7	[6.7-6.8]	9.0	[8.5-9.4]	4.0	[3.7-4.3]
Very high (≥10,000)	6.2	[6.2-6.3]	9.1	[8.7-9.5]	4.3	[4.0-4.6]

CI: confidence interval

The hierarchical logistic regression models including random intercepts for EMS centers did not converge. Table 4 shows the results of logistic regressions fitted with fixed effect models for 1-month survival and neurologically favorable 1-month survival. The odds of being alive at 1 month are lower for females, older patients, cases of arrest of non-cardiac origin and cases in winter or spring. The odds of being alive at 1 month are 5.79 times higher for patients receiving first aid with CPR and AED than for those receiving no first aid. Patients in very high-density areas were significantly more likely to exhibit a better 1-month survival (OR, 1.64; 95%CI, 1.44 -1.87; p < 0.001) and neurologically favorable 1-month survival (OR, 1.47; 95%CI, 1.22 - 1.77; p < 0.001) compared with those in very low-density areas. Even after being adjusted for various factors including individual patient ages, the outcomes were significantly affected by the proportion of neighborhood elderly people (aged ≥65 yrs). That is, patients living in areas where ≥ 25% of people were aged ≥65 yrs had a significantly lower probability of 1-month survival (OR, 0.86; 95%CI, 0.79 - 0.93; p < 0.001) and neurologically favorable 1-month survival (OR, 0.88; 95%CI, 0.78 - 0.98; p = 0.021), compared with those residing in areas where < 15% of people were aged ≥65 yrs.

**Table 4:** Logistic regressions for 1-month survival rates and neurologically favorable 1-month survival rates

	1-month survival					Neurologically favorable 1-month survival				
	
	OR	95%CI			p	OR	95%CI			p
Sex										
Male	1.00					1.00				
Female	0.95	0.90	-	1.00	0.040	0.87	0.80	-	0.93	<0.001
Age (yrs)										
≤59	1.00					1.00				
60 - 69	0.83	0.72	-	0.96	0.011	0.65	0.54	-	0.80	<0.001
70 - 79	0.57	0.49	-	0.65	<0.001	0.32	0.26	-	0.40	<0.001
80 - 89	0.37	0.32	-	0.43	<0.001	0.23	0.18	-	0.29	<0.001
≥90	0.27	0.21	-	0.34	<0.001	0.15	0.10	-	0.22	<0.001
Cause of arrest										
Cardiac	1.00					1.00				
Non-cardiac	0.81	0.78	-	0.85	<0.001	0.49	0.45	-	0.53	<0.001
First aid by bystander										
None	1.00					1.00				
CPR without AED use	1.52	1.45	-	1.60	<0.001	1.99	1.85	-	2.13	<0.001
CPR with AED use	5.73	4.80	-	6.84	<0.001	9.60	7.89	-	11.7	<0.001
Seasons										
Summer (June-August)	1.00					1.00				
Autumn (September-November)	0.95	0.88	-	1.01	0.103	0.94	0.86	-	1.04	0.224
Winter (December-February)	0.84	0.78	-	0.89	<0.001	0.81	0.74	-	0.90	<0.001
Spring (March-May)	0.85	0.80	-	0.91	<0.001	0.80	0.73	-	0.88	<0.001
Population density (/km^2^)										
Very low (<250)	1.00					1.00				
Low (250-999)	1.14	0.99	-	1.30	0.067	1.03	0.85	-	1.25	0.776
Middle-low (1,000-2,999)	1.34	1.18	-	1.53	<0.001	1.29	1.08	-	1.55	0.006
Middle-high (3,000-5,999)	1.53	1.35	-	1.74	<0.001	1.29	1.08	-	1.55	0.006
High (6,000-9,999)	1.63	1.42	-	1.85	<0.001	1.39	1.15	-	1.67	0.001
Very high (≥10,000)	1.64	1.44	-	1.87	<0.001	1.47	1.22	-	1.77	<0.001
The proportion of neighborhood elderly people (aged ≥65 yrs; %)										
<15	1.00					1.00				
15 - 25	0.89	0.80	-	0.98	0.025	0.94	0.82	-	1.08	0.384
>25	0.82	0.72	-	0.94	0.005	0.81	0.68	-	0.96	0.017

CPR: cardiopulmonary resuscitation, AED: automated external defibrillators, OR: odds ratio, CI: confidence interval

## Discussion

Some previous data have suggested an association between population density and survival following OHCA [7-11]. In a previous study of 311 OHCA cases in Kentucky, USA, population density ≥100/square mile was associated with higher survival of OHCA [7]. In one study of 793 OHCA patients in Pennsylvania, USA, survival rates were 9%, 14% and 23% in rural, suburban and urban areas, respectively [9]. Another study of 1,956 patients in Scotland showed no significant difference in survival to discharge between areas with different median response times [11]. However, these studies were conducted in limited geographical areas with relatively small sample sizes. Our study of 101,287 OHCA cases in Japan showed that 1-month survival rates were 5.4% and 9.1% in very low-density (<250/km^2^) and very high-density (≥10,000/km^2^) areas, respectively. To our knowledge, the present study is the first to demonstrate the relationship between population density and survival of OHCA in a nationwide setting. The very large sample size allowed more robust multivariate analyses of survival correlates and precise estimates of odds ratios. In the present study, population density was a consistent independent correlate of 1-month survival and neurologically favorable 1-month survival.

The distribution of EMS centers in different regions of Japan is almost in proportion to population density. The low survival of OHCA in low-density areas is likely to be due primarily to greater call-response intervals in these areas. These findings have important health policy implications for nations that wish to improve survival in cases of OHCA; EMS resource allocation according to population size may cause disparities in response times and subsequent health benefit inequalities between urban and rural areas. Compared to their urban counterparts, rural EMS personnel travel longer distances to provide prehospital EMS, contributing to poor survival outcomes.

Minimizing call-response interval could enhance survival rates. Increasing the number of ambulances could decrease call-response intervals across large, sparsely populated areas, but may not be practical because of costs and a need for cost-effective measures. Nevertheless, health policy makers should make every effort to minimize disparities in EMS availability, and maximize the overall effectiveness of a national EMS system by optimizing the allocation of EMS resources under budget constraints. For example, it may be worth considering helicopter transportation in rural EMS systems [22], though the applicability and effectiveness of implementing this approach would require further investigation [23,24]. The use of other existing resources should also be taken into consideration to optimize call-response intervals. For example, a police first-responder program may be beneficial in regions where ambulance response times are longer [25].

At the same time, other more cost-effective strategies should also be explored. Our study revealed that bystander CPR occurred in only approximately 42% of OHCA cases. While this figure is relatively high compared with many countries [5,26], there is much room for improvement. There is evidence that people may be unwilling to perform this distressing psychomotor task because of fear of doing harm or an aversion to mouth-to-mouth breathing [27].

The present study further corroborated the importance of bystander CPR. Emergency medical professionals should make further efforts to enable the general public to provide CPR when necessary. Although public access defibrillation with AED significantly improved survival, its use was extremely rare. Despite the nationwide dissemination of AED in Japan [6], there was a benefit of AED use in only 0.56% cases of bystander-witnessed OHCA; an analysis of the cost-effectiveness of AED implementation will be essential to evaluate this measure.

Another significant finding in this study was the lower survival rate of OHCA cases in areas with a higher proportion of elderly people. Patients' individual age is a biological factor that definitely influences mortality. Living in an aged society is a social factor that is independent from individual age. Even after adjusting for various factors including individual patients' age and presence of bystander CPR, patients living in aged-population areas had a lower probability of survival following OHCA. This may also be attributed to older patients having greater degree of disease or multiple co-morbidities. However, it could be hypothesized that OHCA patients in aged-population areas may be more likely to receive CPR from elderly bystanders, and the quality of their CPR may be relatively poor, resulting in lower success rates.

Increasing the quality of bystander CPR is a common public health problem worldwide. School-based training programs could be an effective way of training younger populations [28]. New driver's license applicants in Japan are now obliged to undergo CPR training program at driver's school [12]. However, the problem of training the elderly population in CPR remains, especially in rural areas. A previous report showed that the elderly perceived themselves as able to perform and interested in receiving training [29]. Although public health programs that teach CPR to large numbers of the public are expensive, additional emphasis on widespread CPR training for elderly people for improving CPR quality could be worthwhile.

Several limitations of the current study should be acknowledged. First, in common with all registry-based surveys, the validity and integrity of the data are potential limitations, although their effects were likely to be minimized by the large sample size collected with our population-based design. Second, the categories for the population groups were arbitrarily made, and such division could not be generalized to other countries. Third, generalizability of our results could be limited because Japanese geographic characteristics (two-thirds of the country being uninhabitable and a majority of people living in urban areas) could be different compared to other countries. Lastly, our database lacked detailed data to make further risk adjustments for survival; e.g. comorbid conditions of patients or severity of cardiac arrest based on clinical markers, experiences of EMS personnel [30], the existence of specialists of emergency care or cardiologists or treatments available at the receiving hospitals [31].

## Conclusion

Our study is the first to demonstrate in a nationwide setting that population density is a consistent factor independently affecting the likelihood of survival following OHCA events. Appropriate strategies should be implemented to minimize the current disparity in EMS availability and subsequent inequality of health benefits between urban and rural areas.

## Competing interests

The authors declare that they have no competing interests.

## Authors' contributions

HY conceived the study concept and study design and wrote the draft. HY and HM carried out statistical analyses HH performed compilation, synthesis and analyses of data on the geographic information system. TI obtained the Utstein data from the Fire and Disaster Management Agency of Japan on behalf of our research team, and supervised the research project. HY, HM, HH, MA, TO, ST, SK, and TI participated in interpretation of the results and writing of the report. All authors approved the final version.
